# Case Report: No Response to Liposomal Daunorubicin in a Patient with Drug-Resistant HIV-Associated Visceral Leishmaniasis

**DOI:** 10.1371/journal.pntd.0003983

**Published:** 2015-08-25

**Authors:** Nicholas J. Gow, Robert N. Davidson, Rob Ticehurst, Andrew Burns, Mark G. Thomas

**Affiliations:** 1 Department of Infectious Diseases, Auckland City Hospital, Auckland, New Zealand; 2 Department of Infectious Diseases and Tropical Medicine, Northwick Park Hospital, Harrow, England; 3 Pharmacy, Auckland City Hospital, Auckland, New Zealand; 4 Department of General Medicine, Hawkes Bay Hospital, Hawkes Bay, New Zealand; Emory University, UNITED STATES

## Abstract

Visceral leishmaniasis (VL) in patients with HIV co-infection presents a significant therapeutic challenge due to the lessened chance of achieving long-term cure. We report a case of VL in a 60-year-old man with HIV infection who became refractory to anti-leishmania treatment due to multi-drug resistance. In the face of a worsening clinical situation, and with no other options available, he was treated with an experimental regimen of liposomal daunorubicin, which has previously been shown to have *in vitro* activity against *Leishmania donovani* and to be effective treatment of VL in animal studies. To our knowledge, he was the first patient with VL and HIV co-infection to have this treatment evaluated. We report on the lack of response to this treatment and possible causes for its failure.

## Author Summary

In areas where leishmaniasis is endemic, visceral leishmaniasis (VL) is a potentially incurable AIDS illness in HIV infected people. Co-infection with HIV and any of the *Leishmania* species that cause VL can result in failure of the CD4 lymphocyte count to rise above 200 cells/mm^3^ despite sustained, effective suppression of HIV infection, and this failure of CD4 recovery then commonly prevents immune mediated eradication of VL despite aggressive, prolonged anti-Leishmania treatment. The situation is made more challenging by the limited number of agents active against VL and their high rate of adverse effects. We report the first evaluation of liposomal daunorubicin used to treat VL in a patient with HIV and *L*. *infantum* co-infection. We selected this drug on the basis of the proven activity of anthracyclines against *Leishmania* species both *in vitro* and in animal models and the well tolerated use of daunorubicin in HIV infected patients with Kaposi’s sarcoma. This treatment proved unsuccessful in our patient, probably because resistance of *L*. *infantum* to liposomal daunorubicin had been induced by previous treatment with another anti-leishmania drug, miltefosine.

## Introduction

Visceral leishmaniasis (VL) is a relatively common AIDS illness in regions where *Leishmania donovani*, *L*. *infantum* or other *Leishmania* species are endemic, e.g. South Asia, Brazil, Southern Europe [1] and especially East Africa. [2] Between 1986 and 1997, VL occurred in ~ 2% of patients with HIV in Madrid. [3] In patients with severe HIV-related immunodeficiency (CD4 count < 100 cells/mm^3^) the persistence of leishmaniasis commonly prevents adequate CD4 recovery despite attempts to clear VL and consistent suppression of the HIV viral load. [4] Persistent CD4 cytopenia lessens the chance of long-term cure of VL, and co-infected patients with CD4 < 100 at the initiation of VL treatment commonly develop drug resistant VL and die due to progressive VL. [5] New approaches to anti-leishmania treatment are required to reduce morbidity and mortality in patients with HIV infection and VL.

The anthracycline anti-cancer agent doxorubicin has potent activity against *L*. *donovani in vitro* and in animal models of visceral leishmaniasis [6,7] by targeting leishmania DNA topoisomerase enzymes. [8] Encapsulation of doxorubicin within liposomes, to reduce cardiac toxicity, has been shown to provide a dramatic increase in anti-leishmania activity when compared with free doxorubicin in an *in vitro* model of infection. [9] Both doxorubicin and the closely related anthracycline daunorubicin have been relatively well tolerated when used for treatment of Kaposi’s sarcoma in patients with HIV infection. [10] We attempted compassionate-use treatment with liposomal daunorubicin in a patient with well-controlled HIV infection and longstanding VL despite combination anti-leishmania treatment.

## Materials and Methods

**Fig 1:**
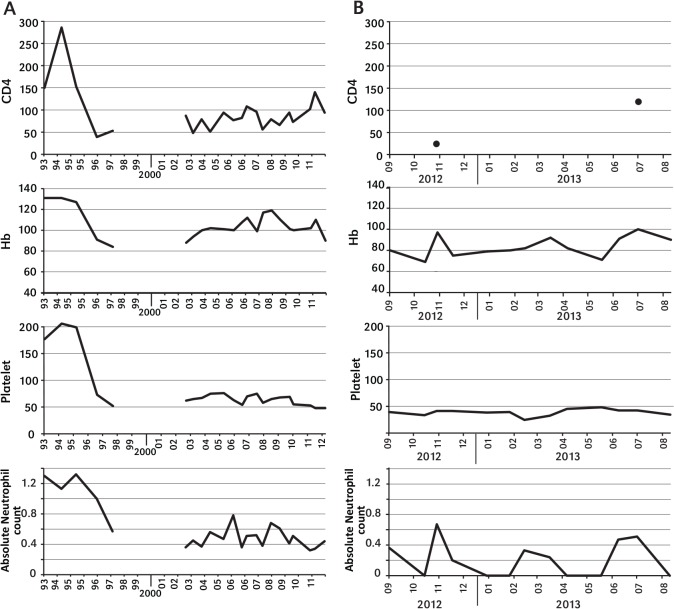
(A) Trend of CD4 count, haemoglobin, platelet count and absolute neutrophil count, prior to treatment with liposomal daunorubicin 1993–2012. (B) Trend of CD4 count, haemoglobin, platelet count and absolute neutrophil count, September 2012-August 2013.

The patient was a 60 year old European homosexual man who had HIV infection diagnosed in 1985. Zidovudine monotherapy was commenced in 1988 and three-drug combination antiretroviral therapy (ART) in 1996. His ART between 2001 and 2013 consisted of lopinavir/ritonavir, lamivudine and nevirapine. He had a prompt sustained response to ART and from 1997–2013 consistently had an undetectable HIV viral load. Despite this, his CD4 count, which had a nadir of 39 in 1996, never rose above 140 despite consistent adherence to his ART (Fig 1A).

In April 1996 he developed drenching sweats and hepato-splenomegaly, and in March 1997 the diagnosis of VL was confirmed by the visualization of amastigotes in bone marrow samples. Infection with *Leishmania infantum* had probably been acquired during a visit to Spain in 1992. Initial treatment with sodium stibogluconate 20 mg/kg for 44 days over 8 weeks achieved a clinical improvement and clearance of parasites from his bone marrow.

Visceral leishmaniasis relapsed in July 1997 and persisted for the next 15 years, despite combination treatment with: paromomycin 15 mg/kg and stibogluconate; stibogluconate, liposomal amphotericin 3mg/kg and gamma interferon 2 megaunits subcutaneously; and since 2000 with 7 days liposomal amphotericin 3 mg/kg daily every 6 weeks, increasing to every 3 weeks in May 2012. Miltefosine 50 mg twice daily, for 28 days every 3 months, was added in March 2005.

*Leishmania* amastigotes were visualized in bone marrow samples collected in September 2004, March, May, and December 2005, November 2006, October 2007, and February 2011; and also in skin lesions biopsied in November 2003, September 2007, and May 2009; lymph nodes biopsied in May 2009 and September 2011; and a sigmoid biopsy in March 2000. An isolate of *Leishmania infantum* cultured from a bone marrow aspirate in October 2007 was tested at the London School of Hygiene and Tropical Medicine in January 2008 and reported to be resistant to stibogluconate (IC_50_ > 60 mg/L) but susceptible to amphotericin B (IC_50_ = 0.3 μM), miltefosine (IC_50_ = 8 μM) and paromomycin (IC_50_ = 4 μM).

Treatment of VL was complicated by significant adverse drug effects including: marked visual impairment presumed secondary to antimonial toxicity; hearing loss presumed secondary to various agents including paromomycin; and multiple episodes of bacteremia arising from the indwelling cannula used for his IV infusions of liposomal amphotericin B. The patient suffered increasing discomfort from his splenomegaly and had an increasing requirement for red cell transfusions. In view of his worsening symptoms and pancytopenia, and the consistent increase in the density of amastigotes in his bone marrow samples despite maximal conventional treatment, alternative therapeutic approaches were discussed at length with the patient and with experts elsewhere.

**Fig 2:**
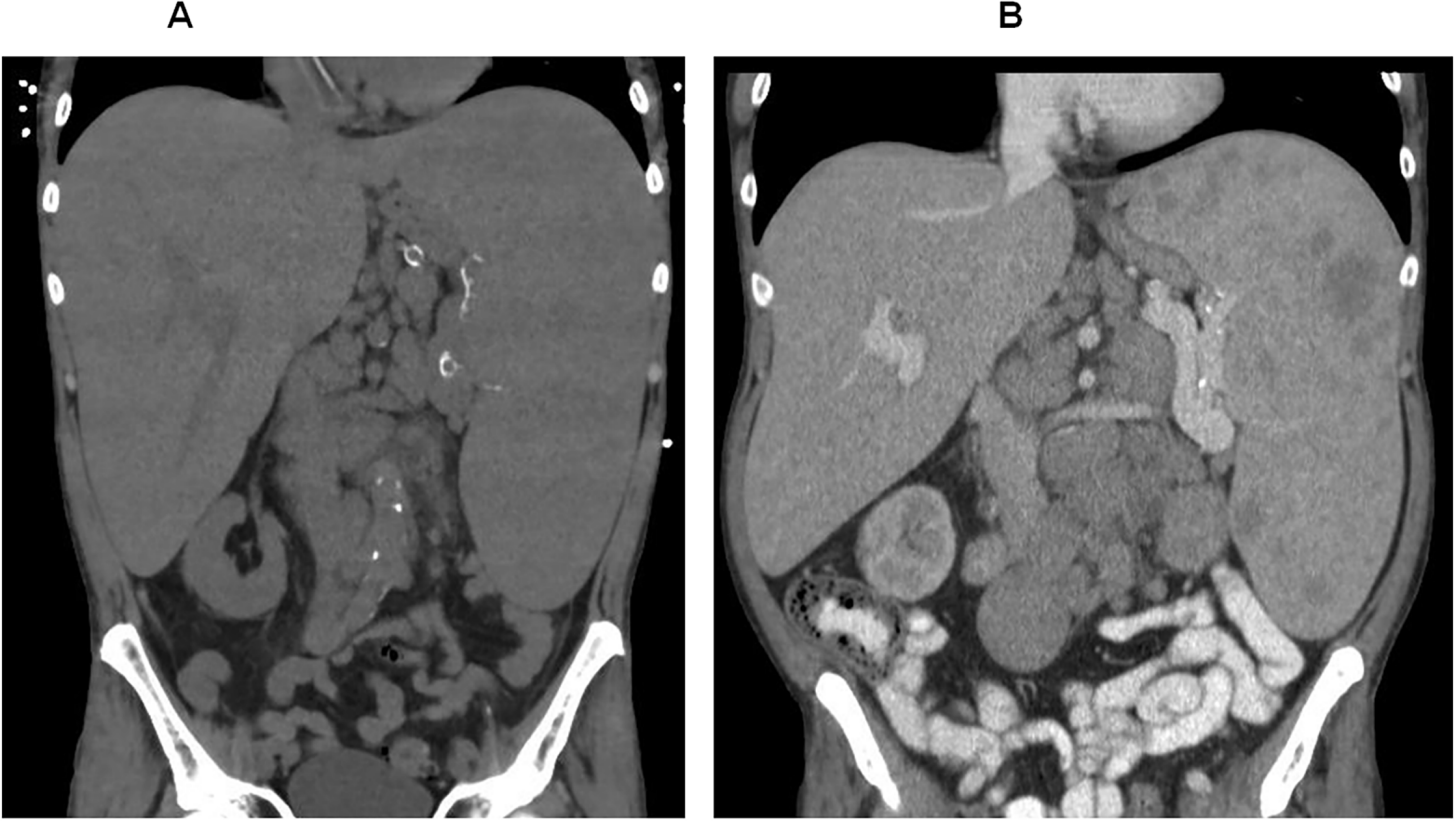
(A) Abdominal computerised tomography (CT) scanning conducted in May 2012. Before treatment with liposomal daunorubicin. (B) Abdominal computerised tomography (CT) scanning conducted in July 2013. After treatment with liposomal daunorubicin, demonstrating no decrease in the patient’s hepato-splenomegaly.

**Fig 3:**
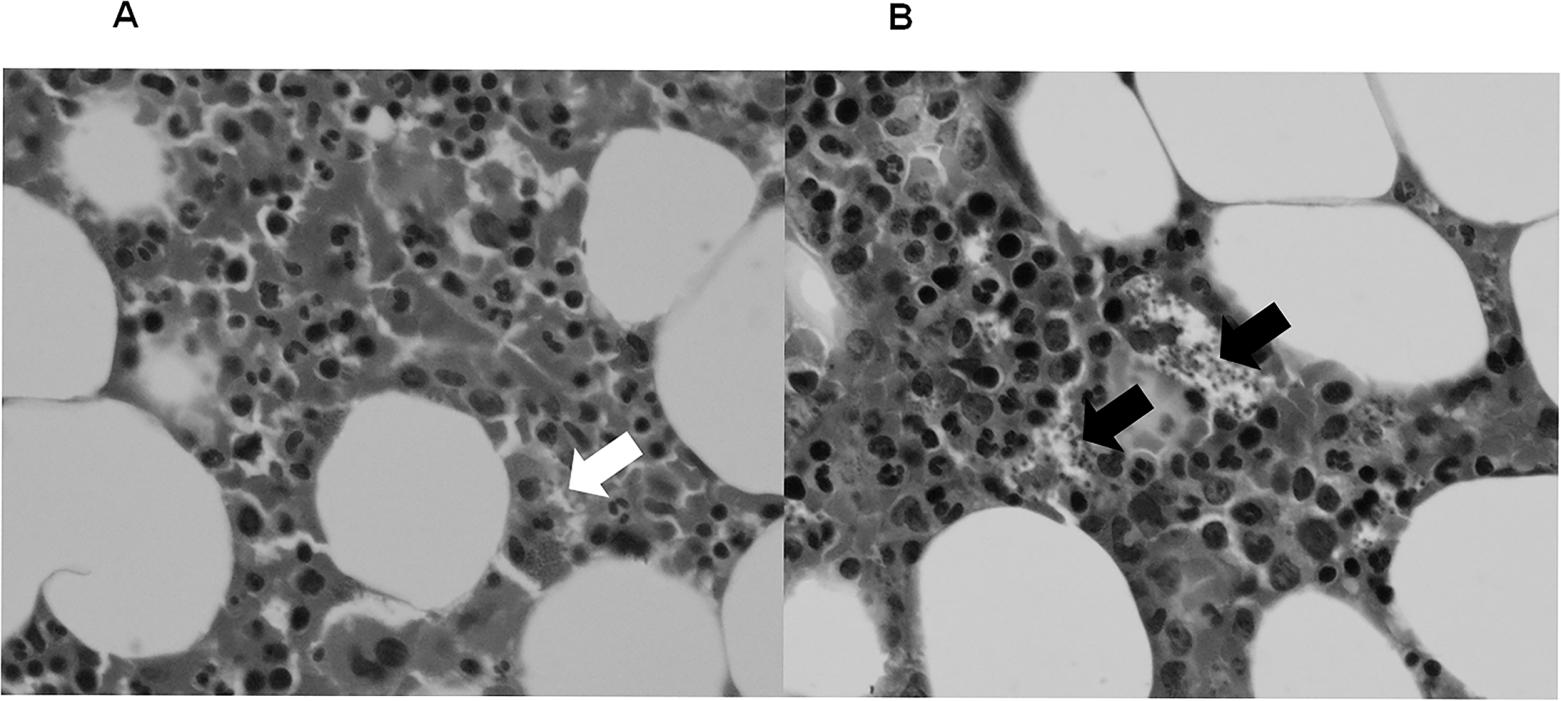
(A) Haematoxylin and eosin stained bone marrow trephines obtained in February 2011 showing occasional amastigotes (white arrow). (B) Haematoxylin and eosin stained bone marrow trephines obtained in February 2013 after liposomal daunorubicin treatment showing a heavy parasite load (black arrows).

With written informed consent from the patient, and agreement from the Hospital Medicines Committee, he received six doses of intravenous liposomal daunorubicin: 34mg on 24 October 2012, 50mg on 7 November, 50 mg on 21 November, 40mg on 7 January 2013, 50mg on 21 January, and 50mg on 4 February. Daunorubicin was chosen instead of doxorubicin because of personal and institutional experience in the use of this agent for the treatment of Kaposi’s sarcoma in patients with HIV infection. The treatment regimen was based on that recommended for the treatment of Kaposi’s sarcoma in patients with HIV infection and mild renal impairment. The patient was carefully monitored for evidence of cardiotoxicity, and other potential adverse effects of daunorubicin therapy. Each treatment resulted in a temporary worsening of his pancytopenia but there were no significant infectious, bleeding or other adverse effects (Fig 1B). There was no decrease in his splenomegaly either on clinical examination or on abdominal imaging (Fig 2). The density of amastigotes in the bone marrow trephine collected 7 days after the last dose of daunorubicin was greater than that in the most recent previous trephine which had been collected in February 2011 (Fig 3). Urine samples collected on May 8 2013, 3 months after the last dose of daunorubicin, showed 3+ agglutination using the KAtex *Leishmania* urinary antigen detection test. In September 2013, 7 months after the completion of the trial of daunorubicin, the patient was in an unchanged clinical condition compared to the immediate pre-treatment period, with persistent abdominal pain due to severe hepato-splenomegaly, pancytopenia and disabling lethargy. The patient decided to cease all treatment of his HIV infection, VL, and other medical conditions. He died of complications of VL within one month of stopping treatment.

### Ethics Statement

The patient provided written informed consent for all procedures (skin biopsy, bone marrow biopsy, and sigmoidoscopy and biopsy) and written informed consent for the trial of liposomal daunorubicin. The Hospital Medicines Committee approved the trial of liposomal daunorubicin.

## Results/Discussion

This is the first reported trial of treatment with liposomal daunorubicin for VL. It was used in a desperately ill patient with HIV infection, CD4 lymphopenia, and 15 years of persistent, drug resistant VL caused by *L*. *infantum*. Despite evidence that liposomes containing the closely related anthracycline agent, doxorubicin, are active against *L*. *donovani*, both *in vitro* and in animal models, we found no evidence of response to treatment with six doses of liposomal daunorubicin (40 mg/m^2^ capped at 50 mg) over a 3 month period.

Potential explanations for the lack of response to treatment in our patient include inadequate concentrations of daunorubicin within infected macrophages and inadequate activity of daunorubicin against the strain of *L*. *infantum* infecting our patient. The dose of liposomal daunorubicin administered to our patient was based on the doses used in trials of the treatment of Kaposi’s sarcoma (40–60 mg/m^2^ every 2 weeks) [10] which have been shown to result in peak plasma daunorubicin concentrations of ~15 μg/ml in HIV infected patients concomitantly being treated with an ART regimen similar to that used in our patient. [11] Liposome encapsulation of doxorubicin resulted in an approximately 50 times enhancement of the anti-leishmania activity of doxorubicin in an *in vitro* model of infection, from increased delivery of the drug to the site of infection within macrophages. [9] Similarly, doxorubicin-loaded nanocapsules have increased anti-leishmanial effect compared to free doxorubicin. [12] However, it is unclear whether these pharmacokinetic advantages were active in our patient, because the uptake of liposomal drugs depends on the phagocytic function of macrophages, which is impaired both in HIV infection [13] and as part of T cell exhaustion in VL. [14] Furthermore, it is possible that daunorubicin is a less potent anti-leishmanial agent than the closely related anthracycline doxorubicin (hydroxyl daunorubicin).

*In vitro* susceptibility testing of *L*. *donovani* amastigotes found that liposomal doxorubicin was active at concentrations (IC_50_ = 9.8 ng/mL) very much lower than those measured in plasma (12 μmol/L) during administration of 75 mg/m^2^ of liposomal doxorubicin. [15] However multi-drug resistant strains of *L*. *donovani* over-expressed an ATP-dependent pump able to export a range of hydrophobic drugs including miltefosine and anthracyclines (daunorubicin and doxorubicin) from the cell cytoplasm. [16,17] While the strain of *L*. *infantum* isolated from our patient in October 2007 was susceptible to miltefosine it is possible that continued treatment with miltefosine resulted in selection of a strain that overexpressed this efflux pump and thus was resistant to anthracyclines. Our repeated attempts to culture *L*. *infantum* from tissue samples between 2007 and 2012 were unsuccessful and thus we were unable to either repeat *in vitro* susceptibility testing or measure the level of expression of the gene for the ATP-dependent pump. Finally, it is possible that the anthracyclines, such as doxorubicin and daunorubicin, may be less active against *L*. *infantum*, which was responsible for infection in our patient, than against *L*. *donovani*, the closely related species which has been used in laboratory evaluations of treatment efficacy. [6–9,12]

In summary, we observed no response to treatment of VL with liposomal daunorubicin in an HIV infected patient. A possible explanation for failure of this treatment was that previous treatment had selected for a multi-drug resistant *Leishmania* strain. Clinicians considering the use of anthracyclines in the treatment of leishmaniasis [18] should be alert to this possibility, and the potential implications for the efficacy of treatment with other anti-leishmanial agents.
